# Development of a Multifunctional Wet Laid Nonwoven from Marine Waste *Posidonia oceanica* Technical Fiber and CMC Binder

**DOI:** 10.3390/polym14050865

**Published:** 2022-02-23

**Authors:** Saoussen Zannen, Mohamed Taher Halimi, Mohamed Ben Hassen, Emad Hashim Abualsauod, Asem Majed Othman

**Affiliations:** 1 Laboratory of Textile Engineering, BP 68 Ksar Hellal, University of Monastir, ISET Ksar Hellal, Hadj Ali Soua, Ksar Hellal 5070, Tunisia; saoussenzannen@hotmail.fr; 2 Department of Administrative Sciences, Applied College, Imam Abdulrahman Bin Faisal University, P.O. Box 1982, Dammam 31441, Saudi Arabia; mthalimi@iau.edu.sa; 3 Department of Industrial Engineering, College of Engineering, Taibah University, Madina 41411, Saudi Arabia; 4 Department of Industrial and Systems Engineering, College of Engineering, University of Jeddah, Jeddah 21959, Saudi Arabia; amothman@uj.edu.sa

**Keywords:** *Posidonia oceanica* fiber, Box–Behnken design, wet-laid nonwoven, thermal conductivity, insulation

## Abstract

A *Posidonia oceanica* waste marine plant was used to produce a wet-laid nonwoven web for multifunction applications. To study the effect of some parameters related to the web characteristics (sheet weight, binder ratio, and pulp ratio) on the mechanical and physical properties of the web, we used a Box–Behnken design plan with three levels. The diagram of the superposed contours graphic method was used to find the optimum parameters of the process for the application of the Posidonia nonwoven fiber on an insulation field. With the measurement of the thermal conductivity properties using the box method, the results demonstrated that the nonwoven fiber from *Posidonia oceanica* marine waste had good insulation properties in comparison with other classical natural fibers (hemp, flax) used in the field of insulation with the big advantage of being a natural product.

## 1. Introduction

In comparison with many synthetic materials, natural fibers are light, inexpensive, abundant, and biodegradable. There is a myriad of literature related to the possibility of using natural fiber in many technical applications. Cotton, hemp, flax, kenaf, agave, halfa, and other natural fibers show a good potential to be used as insulation materials and as reinforcement materials for composites [1,2,3,4].

Posidonia is a marine plant with flowers. This species of seagrass is endemic to the Mediterranean Sea. This perennial loses its leaves in autumn. They are then found, pushed by currents and waves, onto shore. These leaves decompose while curling and form pellets of a thickness of some centimeters called “aegagropiles” [5]. In the Mediterranean region, each summer, the beaches must be cleaned, and this biomass is often removed and not exploited. According to the work by Dural et al. [6], in the Mediterranean Basin alone, total *Posidonia oceanica* production ranges from 5–50 M t per year. One solution to this ecological problem could be the valorization of this available and renewable biomass for the production of environmentally friendly industrial products.

Posidonia exploitation in recent studies has been found to be twofold. For fibers and cellulosic derivates, it can be used as an adsorbent for removing metal-complexed dyes or phenol [6,7,8,9] or for fibers’ and cellulosic derivates’ production [10,11]. The second use [12,13,14,15] is for the preparation of fiber-reinforced composite materials.

In this study, we propose to use *Posidonia oceanica* marine waste plant to produce a dry-laid nonwoven with a 3D structure that can be applied in fields such as insulation, filtration, and packaging. In particular, we plan to apply the design of experiments surface of the response method to optimize the properties of the nonwoven to be utilized in the field of insulation.

## 2. Materials and Methods

### 2.1. Development of the Wet Nonwoven Fabric from Posidonia

Posidonia waste was collected from Hergla beach in Tunisia. It was first prepared manually, then passed through a horizontal opener. One of our main objectives was to use an environmentally friendly and economic process for the separation operation.

Two kinds of fiber were obtained after the opening process:

1. Technical fibers (length greater than 2 mm);

2. Posidonia pulp (length less than 2 mm).

The principle of producing wet nonwovens is very similar to the principle of paper manufacturing. The materials used were Posidonia technical fibers, Posidonia pulp, and a CMC binder. The characteristics of the Posidonia technical fibers and pulp are summarized in Table 1. The carboxymethylcellulose (CMC) binder used in our case has a chemical formula of C_6_H_7_O_2_(OH)_2_CH_2_COONa, a molecular weight of 263.1976 g/mol, and a degree of substitution of 1. CMC is an environmentally friendly polymer material. The nonwoven produced can be considered as a bioproduct.

The characteristics of the different components are summarized in Table 1:

- The average lengths of both the technical fibers and pulp were determined using an optical microscope, Leica, in accordance with the French standard NF G 07-009 (1983);
- The average diameters of both the technical fibers and pulp were measured according to the projection microscope method. This is the reference method for determining the diameter of wool samples in accordance with standard NF G 07-004 (1983). The device used was a Leica-type optical microscope;
- The crystalline index of the fibers was calculated using the equation $Crl\left(\%\right)=\frac{I002-Iam}{I002}\times 100$ [4], where *Crl* (%) is the crystalline index; *I*_002_ is the maximum intensity of the 002 lattice diffraction plane at a 2θ angle between 22°and 23°; *Iam* is the intensity diffraction at an angle 2θ close to 18° representing amorphous materials in plant fibers. Theses parameters were determined using an X-ray diffraction (XRD) device.

A scanning electron microscope (SEM) was employed to determine the cross-sectional view of the Posidonia technical fiber with a magnification of 1000. The microscope used was a TM3000 Hitachi type.

**Figure 1:**
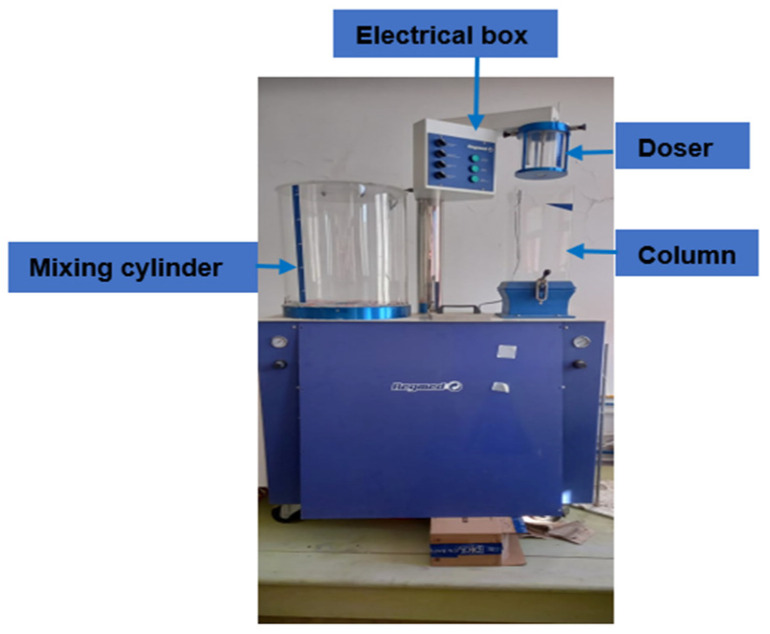
Wet nonwoven machine: Sheet Maker.

The manufacture of the nonwoven fabrics was carried out using an automatic “Sheet Maker” machine (Figure 1).

The process comprises the following four steps:

- Preparation of aqueous suspensions = Phase 1;
- Forming the sheet = Phase 2;
- Dewatering = Phase 3;
- Drying = Phase 4.

This manufacturing process was inspired by the one carried out by Hansen et al. [16]. We attempted to satisfy the major requirements for the manufacturing of a wet-laid nonwoven (web preparation, web forming, web bonding, and drying). In fact, a comparative study was carried out, and all the samples were produced and tested in the same conditions with different phases, detailed as follows.

Phase 1: The fibrous suspensions were prepared in a 50 L-capacity reservoir (mixing cylinder), which made it possible to have a homogeneous suspension by agitation of the water–fiber mixture.

In our case, we used 12 g of fiber mixture of dry Posidonia and its paste, which was suspended in 20 L of water. The concentration of the solution was determined by dividing the mass of the fiber by the total volume of water. In our experiment, C = 0.8 g/L.

The basis weight of the formed sheet is given by the following formula:

M = 0.02657 × G × V.

where:

M = the mass of the material in g;

G = the desired basis weight of the sheet formed in g/m^−2^;

V = the volume of water to be added to the tank in L.

Phase 2: The sheet’s formation involved transferring a volume of 1 L of fibrous suspension from the mixing cylinder to the metering device (or doser). Then, the suspension was delivered to the subsequent stage “formation column of the sail” in a controlled and dosed manner; the suspension distribution was carried out on a metallic sieve.

Phase 3: To separate the fibers from the water in which they were bathing, it was necessary to aspirate the fibers under the fabric by a suction system. This machine was equipped with suction nozzles located underneath the sieve, which ensured the removal of the residual water. Thereafter, the nonwovens were set to dry. The obtained nonwovens were set between two plates and put in an oven for 1 h at a temperature of 105 °C.

Phase 4: After drying, the nonwovens were immersed in a CMC binder solution with a well-defined concentration. Thereafter, the nonwovens were dried at room temperature and subjected to a certain pressure by means of a forming press at a temperature of 150 °C for 90 s. 

**Figure 2:**
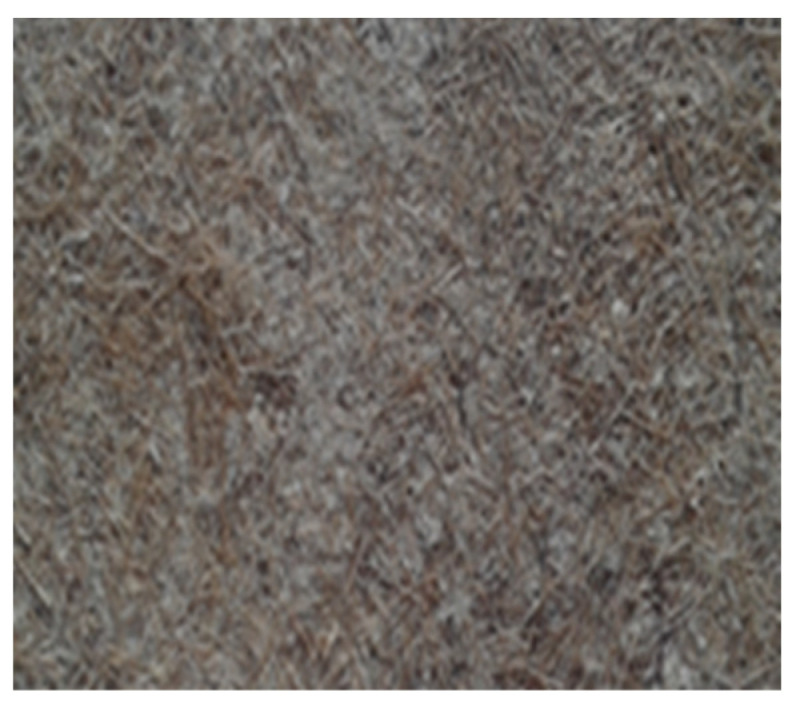
Wet-laid nonwoven Posidonia fiber.

A sample of the nonwovens produced is shown in Figure 2.

### 2.2. Characterization of the Physical and Mechanical Properties of the Wet-Laid Nonwoven

#### 2.2.1. Mechanical Properties

The tensile strength, elongation, air permeability, and thermal conductivity were tested for the nonwoven samples produced. These tests were performed according to the NFG 07-119:2013 standard. Tensile tests were conducted on rectangular samples (150 mm, 50 mm) using a Lloyd Instrument Mers LR 5K dynamometer. The length between clamps was taken at 100 mm, and the speed was set at 100 mm/min.

#### 2.2.2. Air Permeability

Air permeability is an important parameter that directly influences the end-use of the nonwoven fabric, especially in filtration applications. This property, expressed in L/m^2^/s, was measured using a TEXTEST FX 3300 air permeability tester and according to the ISO 9237:1995 standards. The principle of the method consisted of measuring the air flow passing perpendicularly through the test surface with a defined differential pressure for a given time.

#### 2.2.3. Thermal Conductivity

**Figure 3:**
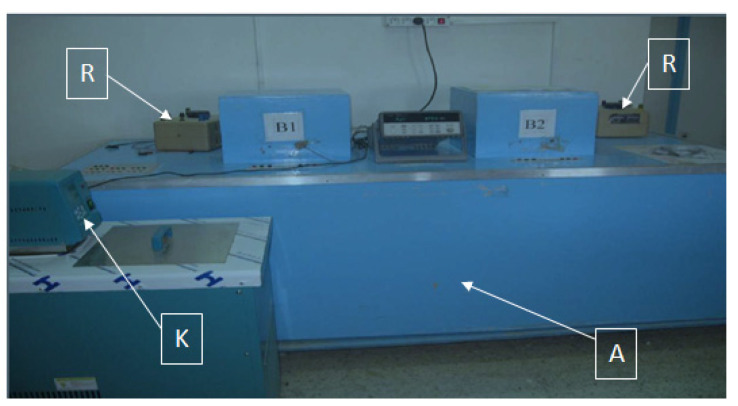
Measurement of the thermal conductivity properties.

The measurement of the thermal conductivity of the nonwoven fabrics, as well as that of the Posidonia fibers was carried out according to the method known as the “box method” [17] (Figure 3).

To measure the thermal conductivity, we used the device shown in Figure 3. It consisted of an isothermal enclosure (A) kept at a temperature lower than the two cans (B1 and B2) using a heat exchanger fed by a cryostat (K). The enclosure (A) constituted the cold source of the system. The heat source was provided by two heating plates, each located in a box fed by a rheostat (R) and containing the samples to be tested. A uniform temperature was imposed in the box through electrical voltage applied to the terminals of the plate. A console indicated the value of this voltage, and an ohmmeter made it possible to measure the resistances of each box. The amount of heat dissipated by the system was then evaluated. Platinum thermostats were used to detect temperatures on the upper and lower surfaces of the sample, and two sensors measured the ambient temperature in the box and the temperature in the room. The thermal gradient imposed between the box (B) and the isothermal enclosure (A) created a heat flow between the upper and lower surfaces of the sample.

### 2.3. Design of Experiments

The nonwoven wet-laid properties were affected by the following [18]:

1. Fibers, pulp, and binder properties P1;
2. Manufacturing process parameters P2;
3. Web characteristics (web density, fiber, pulp, and binder ratio) P3.

Because we aimed to develop a new product, we opted to fix P1 and P2 and to vary P3. For the web characteristics, three factors were studied:

1. Sheet weight (g/m^2^);
2. Percentage of binder (%);
3. Percentage of pulp (%).

To study the effect of the web characteristics on the strength and on the physical and mechanical properties of the nonwoven, we used a Box–Behnken surface design plan with 3 factors and a total of 15 experiments. A Box–Behnken design is a plan in which all the design points are at the center of the design and centered on the edges of the cube, equidistant from the center. Key features of this design are as follows:

1. It allows efficient estimation of quadratic terms in a regression model;
2. It usually consists of fewer design points and, therefore, is less expensive to run than central composite designs.

Our regression model can be estimated by the following Equation (1):

Y = b_0_ + b_0_ X_S.W_ + b_2_ X_B_ + b_3_ X_p_ + b_11_ X^2^_S.W_ + b_22_ X^2^_B_ + b_33_ X^2^_P_ + b_12_ X_S.W_ X_B_ + b_13_ X_S.W_ X_P_ + b_23_X_B_X_P_ (1)

where Y is the response of the dependent variable (air permeability, strength at break and elongation); X_S.W_, X_B_ and X_P_ are the normalized values of S.W, B, and P, respectively; b_0_, b_i_, and b_ij_ are unknown characteristic constants estimated from the experimental data.

Table 2 and Table 3 show the levels of different factors and the absolute and normalized values obtained for the independent variables in the 15 tests required to construct the model.

We chose the ranges of the pulp and binder percentage according to our research based on good practices for nonwoven mills [17].

After the preliminary study, we found that a maximum sheet weight of 30 g/m^2^ could be obtained on our machine. When the sheet weight was less than 15 g/m^2^, the cohesion of the sheet was very low; the obtained nonwoven could not be manipulated at later stages.

## 3. Results and Discussion

Values of the coefficients for the different dependent variables are presented in Table 4. R^2^ is the coefficient of determination of our models.

### 3.1. Influence of Control Factors on the Properties of the Nonwovens Studied

The analysis of variance is presented in Table 5. We accepted the hypothesis H_0_ if P is less than 0.05.

H_0_: the factor has a significant effect on the response with a probability P.

**Figure 4:**
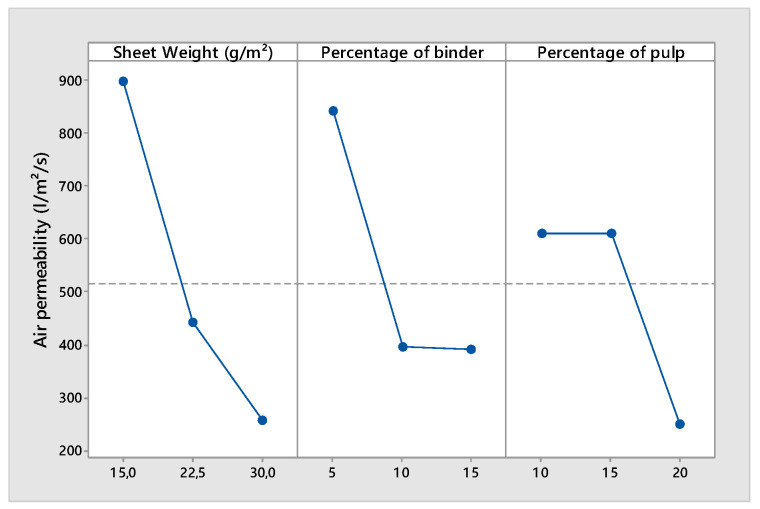
Main effects plot for air permeability.

According to Table 5, the main factors SW, P, and B and the interaction of Level 2 between SW and P had a significant effect on the air permeability. When the weight of the sheet was multiplied by two, the permeability of the nonwoven decreased by more than 70% (Figure 4).

This indicates that depending on the final application, the choice of a good weight is critical.

For filtration (for example), it would be more appropriate to use a sheet with a low weight.

Nonwoven fabrics are complex structures composed of fibers arranged in random directions. Based on the theory of Darcy, Kozeny, and Carman, many experimental and theorical models have been established to evaluate the permeability of nonwovens. It was demonstrated that the permeability of nonwovens depends on the fiber diameter, pore shape and size, and porosity of the structure [19].

In particular, the air permeability of the nonwoven fabric decreased when the thickness increased and increased when the porosity increased. An increase in the binder ratio negatively affected the porosity and the permeability of the structure, while the binder, which is part of the carboxymethylcellulose (CMC) family, coated the structure of the web and decreased the porosity.

**Figure 5:**
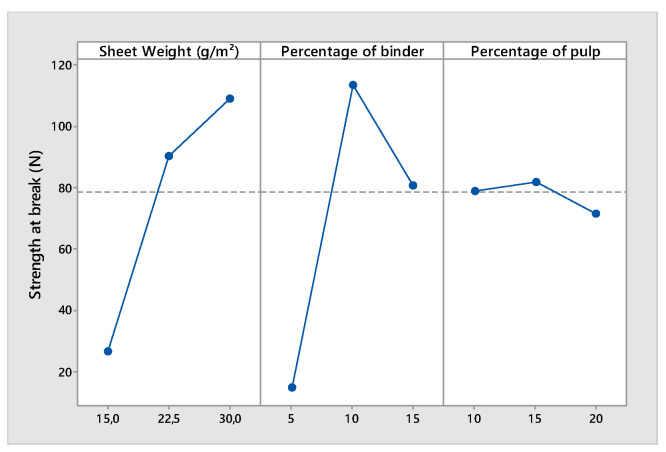
Main effects plot for strength at break.

**Figure 6:**
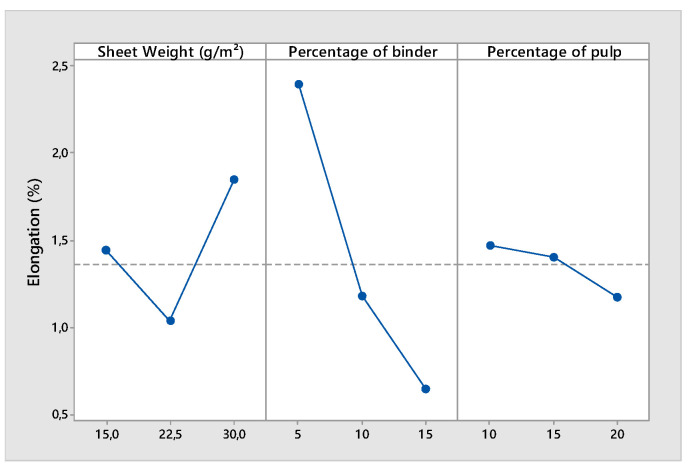
Main effects plot for elongation at break.

The same phenomenon was observed when a high ratio of pulp was used. The pulp was formed by a small fiber with a low diameter and length. A high percentage of pulp affected the average pore size and diameter of the structure and had a negative effect on the permeability. It is interesting to remark that there was a positive interaction between the sheet weight and the ratio of the pulp. The effect of the percentage of the pulp on the permeability was different for a high web weight than for a low sheet weight. For a high web weight (thick nonwoven), the permeability of the structure depended more on the pore sizes and shapes. For a low sheet weight (thin nonwoven), the average fiber diameter (decreased by the addition of a high percentage of pulp) would affect the permeability of the structure more. Figure 4, Figure 5 and Figure 6 demonstrate that the mechanical properties of the nonwoven depended on the weight of the sheet and the binder ratio. In contrast to the woven and knitted structure formed by yarns with good mechanical properties, the nonwovens were composed of a fiber structure. The wet-laid nonwoven’s mechanical properties are given by inter-fiber friction and the binder percentage and properties. The more the quantity of fibers and binders increased (sheet weight increased), the more the strength increased. The effect of inter-fiber friction was more important for thick webs than for thin webs, contrary to the effect of the binder ratio. For thin webs, the binders influenced the resistance of the nonwoven mor than the inter-fiber friction. This explains the influence of the interaction between the web thicknesses and the ratio of the fiber to binder (Table 5).

### 3.2. Optimization of the Nonwoven Manufacturing Process Using the Graphic Method for a Specific Application

Since the nonwoven produced can be used in many fields with different types of performance required, we must select an application and then optimize the parameters of the manufacturing processes. One big advantage of Posidonia fibers is their fireproof property. We decided to orient our application to an insulation product. The availability of the results for other natural fibers in the literature helped us compare our product to existing structures on the market.

Thus, we adopted the superimposed contour diagram method to determine the optimal case. The principle of this graphical method consists of giving for each answer an objective and an acceptance zone. Then, we graphically sought the zones that met the criteria. In this method, we set one of the three parameters, which was the sheet weight, to 22.5 g/m^2^ and varied the other two to find the isoresponse diagrams for each property. Then, a study of the optimization was performed according to a pre-established experimental database, while considering the choice of the application. Thus, the choice of the interval was made in order to obtain:

1. Low air permeability;
2. Important strength and elongation.

**Figure 7:**
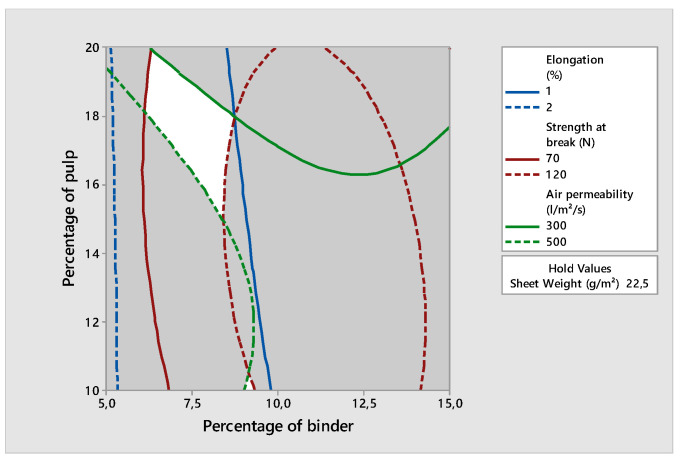
Diagram of the superposed contours.

This diagram of the superimposed contours was produced using the MINITAB software. This made it possible to draw the different contours of each studied parameter and to implement a compromise zone where each point validates the optimal conditions proposed. Therefore, the choice of the optimal condition was made in this white area, as shown in the diagram. From the diagram in Figure 7, we chose:

- Sheet weight: 22.5 g/m^2^;
- Percentage of binder: 7.5%;
- Percentage of pulp: 18%.

### 3.3. Characterization of the Nonwoven Obtained under Optimal Manufacturing Conditions

By proceeding under optimal manufacturing conditions, nonwovens were produced. Subsequently, the different characteristics of these new materials were determined according to the standards, and they are illustrated in Table 6.

The measurement of thermal conductivity was repeated five times for both structures: nonwoven and fibers. The thermal conductivity of the Posidonia fibers and the nonwoven fabrics compared to those of some other materials is illustrated in Table 7.

From this table, we can note that the Posidonia nonwoven had a lower thermal conductivity than that of the Posidonia fibers. This is equivalent to stating that the nonwoven has a greater thermal resistance than that of the fibers.

In fact, similar results have been found with hemp fibers whose thermal conductivity varies from 0.04–0.06 W·m^−1^·K^−1^, while needle-punched nonwovens based on these fibers have a lower thermal conductivity ranging from 0.028–0.04 W·m^−1^·K^−1^ [25]. This can be explained by the structure of the material, which influences their properties.

Compared to other materials, the conductivity of the Posidonia fibers, as well as the materials at its base is very interesting since it was in the conductivity zone of materials classified as insulators such as polyurethane, mineral wool, perlite, and vermiculite. The conductivity of Posidonia-based nonwovens was close to that of air (*λ* = 0.024–0.026 W·m^−1^·K^−1^), which is the best thermal insulation. This important insulation characteristic of Posidonia fibers is due to several intrinsic properties of this biomass. Indeed, it is known that the thermal conductivity of the materials is greatly influenced by their physical structure. Thus, crystalline materials are better heat conductors than amorphous materials [25]. By examining the crystallinity index of Posidonia fibers determined in a previous work [26], it can be seen that the crystalline zones represent only 30% versus 70% for amorphous fibers. As a result, the amorphous zones in this structure are larger than those in crystalline materials, hence the low thermal conductivity.

**Figure 8:**
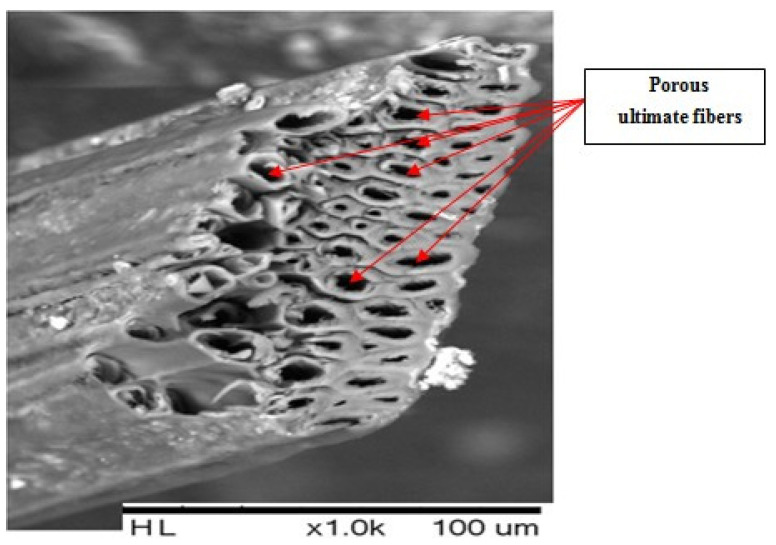
Cross-sectional view of a raw Posidonia technical fiber.

In addition, the thermal conductivity of the materials increased by their porosity. The more porous the material, the lower its thermal conductivity is [27]. By observing the structure of the Posidonia fiber reported in earlier research [28], we found that it was porous, and we show the SEM images in cross-section of the fiber in Figure 8.

Then, the porosity and the amorphous structure of the Posidonia fibers explain well the good thermal insulation of this biomass. This further confirmed the possibility of using these materials as thermal insulation.

## 4. Conclusions

Posidonia is a marine plant with flowers. In the Mediterranean region, every summer, the beaches must be cleaned, and this biomass is often removed. According to some researchers, total *Posidonia oceanica* production ranges from 5–50 M t per year.

The *Posidonia oceanica* marine plant was used to produce a wet-laid nonwoven web for multifunctional applications. To study the effect of some parameters related to the web characteristics (sheet weight, binder ratio, and pulp ratio) on the mechanical and physical properties of the web, we used a Box–Behnken design plan with three levels. The results demonstrated that the web weight (sheet weight) was the most important parameter that affected the mechanical properties and permeability of the nonwoven. The percentage of binders also had an important effect, and finally, the percentage of pulp affected the air permeability more.

We found also that some interactions were non-negligible, for example the cross-effect between the web weight and the ratio of pulp. The effect of the percentage of pulp on the permeability was different for a high web weight than for a low web weight. For a high web weight (thick nonwoven), the permeability of the structure depended more on pore sizes and shapes.

For a low web weight (thin nonwoven), the average fiber diameter (decreased by a high percentage of pulp) would affect the permeability of the structure more.

We decided to use a diagram of the superposed contours graphic method to find the optimum parameters of the process for the application of a Posidonia nonwoven in the insulation field.

After the measurement of the thermal conductivity properties using the box method, the results demonstrated that due to the porosity and the amorphous structure of the Posidonia fibers, nonwovens from *Posidonia oceanica* marine waste have good insulation properties in comparison with other classic natural fibers (e.g., hemp, flax) used in the field of insulation with the potential to be a natural product.

The multifunctional nonwoven produced can be used in many other technical fields: composites, agriculture, the food industry, the automotive industry, packaging, and filtration.

## Figures and Tables

**Table 1:** Characteristics of the nonwoven component.

Component	Average Length (mm)	Average Diameter (µm)	Crystallinity Index (%)
Technical fiber	5	165.54	31.19
Pulp	0.5	20.35	49.91

**Table 2:** Levels of the Box–Behnken design.

	Levels
Factors	−1 0 1
Sheet weight (g/m^2^)	15 22.5 30
Percentage of binder (%)	5 10 15
Percentage of pulp (%)	10 15 20

**Table 3:** Absolute and normalized values of the operational variables.

Test	S.W (g/m^2^)	B (%)	P (%)	X_S.W_	X_B_	X_P_
1	15.0	15	15	−1	1	0
2	22.5	10	15	0	0	0
3	22.5	5	10	0	−1	−1
4	22.5	10	15	0	0	0
5	30.0	5	15	1	−1	0
6	15.0	10	20	−1	0	1
7	30.0	15	15	1	1	0
8	22.5	5	20	0	−1	1
9	30.0	10	10	1	0	−1
10	15.0	5	15	−1	−1	0
11	22.5	15	20	0	1	1
12	22.5	10	15	0	0	0
13	22.5	15	10	0	1	−1
14	30.0	10	20	1	0	1
15	15	10	10	−1	0	−1

**Table 4:** Values of the coefficients for the different dependent variables.

Coefficients and Statistical	Air Permeability	Strength at Break	Elongation
parameters	(−l L/m^2^/s)	(N)	(%)
b_0_	404.2	137.2	0.828
b_1_	−319.8	41.32	0.204
b_2_	−225.6	32.90	−0.874
b_3_	−180.8	−3.70	−0.150
b_11_	140.0	−28.21	0.631
b_22_	220.3	−68.61	0.381
b_33_	−153.2	−13.29	−0.009
b_12_	−170.9	+23.21	0.121
b_13_	288.7	−33.75	0.300
b_23_	79.7	−14.88	−0.127
R^2^	0.9430	0.969	0.791

**Table 5:** Analysis of variance.

Air Permeability (L/m^2^/s)				Strength at Break (N)			Elongation (%)		
	DF	F	P	DF	F	P	DF	F	P
S.W	1	29.18	0.003	1	43.39	0.001	1	0.70	0.440
P	1	9.33	0.028	1	27.51	0.581	1	0.38	0.566
B	1	14.52	0.012	1	0.35	0.003	1	12.85	0.016
S.W × P	1	11.89	0.018	1	14.47	0.013	1	0.76	0.424
S.W × B	1	4.17	0.097	1	6.85	0.047	1	0.12	0.74
P × B	1	0.91	0.385	1	2.81	0.154	1	0.14	0.728
S.W × S.W	1	2.58	0.169	1	9.33	0.028	1	3.10	0.139
P × P	1	3.09	0.139	1	2.07	0.210	1	0.00	0.980
B × B	1	6.39	0.053	1	55.20	0.001	1	1.13	0.337

**Table 6:** Properties of the nonwovens obtained by the wet process under optimal manufacturing conditions.

Properties	Optimal
Nonwoven Air permeability (L/m^2^/s)	581.5 ± 34.12
Tensile strength (N)	80.65 ± 8.34
Elongation (%)	1.75 ± 0.15

**Table 7:** Thermal conductivity of Posidonia fibers, nonwovens, and other materials [20,21,22,23,24].

Material	Thermal Conductivity *λ* (W·m^−1^·K^−1^)
Posidonia fiber	0.038 ± 0.002
Optimum Wet laid Nonwoven	0.0291 ± 0.0016
The air	From 0.024 to 0.026
Polyurethane	0.028
Hemp	From 0.04 to 0.06
Flax	From 0.04 to 0.046
Mineral wool	0.04
Vermiculite	0.058
Perlite	0.065
Wood and derivatives	From 0.12 to 0.17
Glass	1
Reinforced concrete	1.7
Natural stones	From 1.4 to 2.91
